# Organ Transplants from Living Donors – Halachic Aspects^*^

**DOI:** 10.5041/RMMJ.10042

**Published:** 2011-04-30

**Authors:** Mordechai Halperin

**Affiliations:** Senior Researcher, the Schlesinger Institute for Medical Halachic Research, Shaare-Zedek Medical Center, Jerusalem, Israel; Chief Officer of Medical Ethics, Israeli Ministry of Health; Chief Editor of “Assia”; Co-editor of “Jewish Medical Ethics”

**Keywords:** Organ donation, organ transplantation, living donor, Jewish law, halacha

## Abstract

This manuscript is a survey of the halachic attitudes toward organ transplant procedures from a living donor which can be defined as life-saving procedures for the recipient or at least life-prolonging procedures. Three fundamental problems concerning the halachic aspects of such transplantation are discussed in detail: the danger to the donor, donation under coercion, and the sale of organs and tissues. The terms “halacha” and “Jewish law” are defined in the introduction.

## INTRODUCTION

The halachic discussion of ethical dilemmas in medicine is ancient, its principles interspersed throughout the Bible and the Talmud. Over all historical periods halachic literature has included discussions on medical topics.

Before any discussion on the *halachic* aspects of transplantation from living donors, the terms *halacha* and *Jewish law* must be defined and understood.

Halacha includes those issues of halachic rights and obligations as well as issues of religion and morality – in other words, those commandments that deal with interpersonal relationships as well as with the relationship between man and God. This category is broader than “Jewish law”.

Jewish law is a modern concept that includes those issues in halacha that are the subjects of legal rights and obligations. Such subjects are considered legal issues under other legal systems, as opposed to religious and moral issues which are not. If a somewhat simplified definition will suffice, then we may say that Jewish law deals principally with interpersonal laws and almost not at all with the laws between man and God. The obligations of the physician, the patient, and society, for example, belong in Jewish law, but the laws on practicing medicine on Shabbat or on Yom Kippur do not. They belong, however, in the broader field of halacha.

There are three fundamental problems concerning the halachic aspects of transplantation from living donors: 1) The danger to the donor; 2) donation under coercion; and 3) sale of organs and tissues.

We shall discuss here only transplants which can be defined as life-saving procedures for the recipient or life-prolonging procedures. In halachic terms these are cases of *Pikkuach Nefesh*. It should be emphasized that where a procedure is not a life-saving measure, all halachic authorities agree that one may not significantly endanger a donor’s life.

## 1) DANGER TO THE DONOR

### THE COMMANDMENT OF LIFE PRESERVATION

Everyone is obligated to try to save his own life or the life of another who is in danger. There are two aspects to this commandment: the preservation of life and the restoration of “lost property”. The commandment of the preservation of life is derived from the verse: “You shall therefore keep my statutes and my judgments: which if a man do, he shall live by them” (Leviticus 18:5). From this the sages deduced: You shall live by them, but not die by them.^1^ This implies that preservation of human life is the essential purpose of the commandment. Since the Torah clearly conveys this idea, there is no doubt that one must make every effort to save life.

The preservation of life overrides all but three prohibitions of the Torah: idolatry, illicit sexual intercourse, and the shedding of blood.^2^–^4^ Thus if it is necessary to set aside the Sabbath laws, to eat on the Day of Atonement, or to suspend other commandments in order to save human life, the Torah obligates us to save that life since this takes precedence over all the commandments of the Torah, except for the three mentioned. Thus, if one is confronted with the choice of killing one’s fellow man or being killed oneself, the Torah calls for sacrificing one’s own life rather than killing another. The reason for setting aside most commandments is the prevention of death. If a life will be lost in any case, the justification for violating the commandment prohibiting killing is nullified.^5^

Suicide is forbidden as part of the prohibition of killing.^6^ Thus, suicide is prohibited, even when it is intended to save the life of another. It follows that one may not permit removal of a vital organ, even if the donor were to consent.

Halacha is clear in two cases. If Mr A’s life is in danger and Mr B can save Mr A without endangering his own life, he must do so. If Mr B can only save Mr A by sacrificing his own life, he may not do so (unless such a step has to be taken under the special rules of war: in war one is obligated to endanger oneself to save others; therefore it is halachically prohibited to abandon a battle-field^7^,^8^).

What would the ruling be in a case where Mr B can save Mr A’s life by endangering, but not necessarily sacrificing, his own life? It would seem that the possibility of saving Mr A’s life should outweigh other considerations and *require* Mr B to risk his own life. Indeed there is support for such a ruling in the Palestinian Talmud.^9^–^11^

However, this opinion finds no acceptance by the halachic authorities of generally accepted codes. This led Rabbi Joseph Karo to conclude that the acknowledged authorities held that the Babylonian Talmud disagrees with the view of the Palestinian Talmud on this point.^12^ Thus there is no halachic obligation to risk one’s own life in order to save another.^13^ As this opinion is derived from the Babylonian Talmud, it is definitive.^14^–^16^ Furthermore, other authorities held that just as one may not sacrifice one’s own life to save that of another, so one *may not risk* one’s life to save that of another.^17^

### THE INFINITE VALUE OF HUMAN LIFE

The concept of the infinite value of human life may be the basis of a law formulated in the Tosefta.^18^ Maimonides^19^ accepted the opinion of the Tosefta and the Palestinian Talmud^20^ according to which it is prohibited to kill an individual human, even to save the lives of several others.

The basis of this law may be very interesting. According to some Jewish scholars, it is rooted in the infinite value of each human life. This value cannot be measured on any ordinary scale.^21^–^23^ Thus, similarly to the well known principle of set theory, that there are no fewer points in a line of length *a* than in a line of length *2a*, one has no right to say that the value of an individual life is less than that of a group.

Although the principle which prohibits the risking of one’s life to save that of another could be taken to absurd lengths, the halachic authorities emphasize that one may, and indeed one must, undertake a “reasonable” risk to save the life of another.^24^–^28^ Unfortunately, the definition of the acceptable level of risk has not been formulated. One guideline is clear. A risk such as one might normally take in everyday activity or in the course of earning a living is considered acceptable. Such an acceptable risk offers no justification for refraining from saving a life.^24^,^29^,^30^

This guideline calls for a clarification of the level of risk in donating blood, skin, bone-marrow, or a kidney. At one end of the spectrum we have blood donations which are associated with a minimal level of danger and discomfort. The conclusion is clear: a donor is halachically obligated to give blood to save another’s life. At the other end of the spectrum is the procedure of kidney transplantation. Although this does not immediately endanger the life of the donor, there was a long-standing controversy among physicians regarding the long-term damage resulting from removal of a kidney to the health of the donor.^31^,^32^ if there is a high probability of shortening the life of the donor, the removal of his kidney might be prohibited. An act which shortens life is as much an act of killing as one which leads to immediate death;^33^ thus shortening life is clearly prohibited (see also reference 34).

Even if the probability of death or shortening life is not high, the suffering resulting from the surgery and during recovery may be substantial. It follows that even when donating a kidney for life-saving purposes is not prohibited, it is not obligatory.

### MAY ONE SACRIFICE A LIMB TO SAVE A LIFE?

The situation in which Mr A can save Mr B’s life by Mr A’s sacrificing one of his limbs was discussed by *Radbaz* who ruled that Mr A is under no obligation to do so,^35^ based on a verse in Proverbs^36^ (cf. the Babylonian Talmud^37^–^39^).

Although Mr A is not obligated to sacrifice a limb to save Mr B’s life, he may choose to do so. Moreover, the rabbis encouraged saving the life of another even at the cost of sacrificing one’s own limb. Mr A’s choice to sacrifice a limb and thereby save Mr B is clearly a mitzvah.

There is some similarity between the case discussed by *Radbaz* and our subject. In donating a kidney, one sacrifices an organ in order to prolong the life of another. Furthermore, in contrast to removal of a kidney which does not cause disability, removal of a limb *does* cause a substantial disability.

### CONCLUSIONS OF SECTION 1:

- A donor who gives a kidney in order to prolong the life of another or to improve his quality of life fulfills a mitzvah of great merit.
- Nevertheless, even though the donor’s life is not thereby shortened, there is no halachic obligation to donate a kidney.^40^–^43^

## 2) DONATION UNDER COERCION

### THEFT AND PERSONAL INJURY

May a patient attempt to save his own life by compelling another to donate an organ? Consider, for example, a patient with a rare blood type who is injured and whose life depends on an immediate blood transfusion. The blood bank does not have the critical blood type available but knows of a suitable donor. That potential donor refuses to donate blood, even though this means that the patient will die. May the potential donor be compelled to give blood?

Related questions arise when the potential donor is legally incompetent to give consent to the procedure. A retarded or autistic donor, or a donor who has not yet reached the age of majority, is legally incompetent to consent to any procedure, and the guardian’s consent is valid only when it is in the interests of the ward. May such an incompetent person be accepted as a donor of blood in order to save the life of the injured patient?

There are two halachic prohibitions in drawing blood from a donor without his consent: 1) The prohibition of “theft”; and 2) the prohibition of injuring a person.

The prohibition of “theft” is derived from the verse: “Thou shalt not steal”,^44^,^45^ and it includes doing any damage to another person or his property.^46^ Drawing blood without legal permission is accordingly an act of theft. Similarly it is forbidden to injure another without justification.^47^,^48^ Although these procedures are in general prohibited, they may be permissible if intended for life-saving purposes.

### PRESERVATION OF LIFE VERSUS “THEFT” OR INJURY

The preservation of life overrides all but three prohibitions of the Torah (idolatry, illicit sexual intercourse, and the shedding of blood). One might thus conclude that the prohibition of theft and injury to others are suspended in life-saving situations.

Accordingly, it might appear that one may save one’s life by compelling a suitable donor to give blood, just as one may save one’s life by eating on the Day of Atonement, by setting aside the Sabbath laws, or by eating otherwise prohibited food.

Nevertheless, in cases of theft or injury to another, a second party is involved, and the circumstances are therefore not comparable. Discussions of similar point appear in the Talmud, the works of early and more recent authorities.^43^,^49^–^61^

### KILLING AND “ACTS RELATED TO KILLING” *(ABIZRAIHU)*

Most authorities hold that the saving of life does not supersede any “act related to killing” – *abizraihu*.^62^,^63^ (On the other hand, according to Maimonides,^64^ only the actual violation of the three severe prohibitions calls for sacrificing one’s life. Unlike Ran, Maimonides does not extend the obligation to sacrifice one’s life to include acts related to the three prohibitions (cf. Tosafot^65^)).

Rabbenu Yonah^66^ has stated further that personal injury which causes severe psychological suffering may be considered as an “act related to killing”; therefore it would not be suspended even in life-saving situations. Consequently, the coercion of potential blood donors might still be held to be prohibited even in life-saving situations.

### SUMMARY OF SECTION 2:

The question of coercing a donor to donate an organ or body tissue in order to save the life of another is not simple. Its solution depends on a number of fundamental factors:

- Does the preservation of life supersede all but three prohibitions?
- Is personal injury an act “related to killing?”
- If so, does the preservation of life supersede acts related to killing?

These three questions are the subject of ongoing controversy among halachic authorities. Nevertheless, a patient *may not* attempt to save his own life by compelling another to donate an organ.

## 3) SALE OF ORGANS AND TISSUES

The issue of sale of organs and tissues is a sensitive one; the emotional aspects of the issue cannot be neglected in the discussion. Nevertheless, the halachic aspects of the issue must be discussed dispassionately in the light of authoritative sources. Here we shall deal with the halacha pertaining to the sale of human hair, blood, and kidneys.

### SALE OF HAIR AND KIDNEYS

The Mishnah^67^ mentions the sale of hair as a legitimate way of raising money. Rabbi Akiva said: “You must fulfill your financial obligations even if you have to sell the hair upon your head to do so!” The Babylonian Talmud^68^ states that sale of hair is a legitimate method of raising money. The Palestinian Talmud relates that Rabbi Akiva’s wife sold her braids to support her husband who was studying Torah.^69^ This indicates that the human origin of biologic tissue does not necessarily disqualify it from sale.

One might say that there is no essential difference between the sale of hair intended for a wig and skin intended for grafting onto the head of another. But one might distinguish between the procedure of cutting the hair, which is permitted, and the procedure of removal of a donor’s skin, which might be considered to be injury and thus prohibited. Moreover, hair regrows as contrasted with organ or tissues. This brings us to the basic question of a person’s right to injure himself.

### INJURING ONESELF

All authorities agree that it is prohibited to injure oneself irreversibly.^70^ There is a division of opinion among contemporary authorities regarding the question whether a person is considered to *own* his body. According to Rabbi Shlomo Yosef Zevin a person does not own his body.^71^ Rabbi Saul Israeli, on the other hand, is of the opinion that a person does own his body (see the addenda to Rabbi Zevin’s article).

This is derived from the principle that wanton destruction is not permissible.^72^ According to Rabbenu Yona, the Torah prohibits unnecessary spending of money^73^ (cf. Maimonides, reference 74). But Maimonides wrote that the rabbis prohibited unnecessary spending.^75^ This would seem to mean that the Torah does not prohibit it. There is also a division of opinion regarding the status of the prohibition to injure oneself. According to Meiri^76^ the rabbis prohibit injuring oneself. But Rashba^77^ wrote that the Torah prohibits this.^78^

There are differences of opinion among the sages in cases where one “injures” oneself for beneficial effects. According to one Talmudic source^79^ a person may injure himself for a beneficial purpose, just as one may destroy one’s own tree or any other property for beneficial purposes.^80^ In another source^81^ we find a rather different opinion, according to which one may not injure oneself for “minor” benefit,^82^ while this would be permissible in order to achieve “great” benefit.^83^ According to this opinion, financial profit would be considered “minor”, while avoidance of pain and suffering, on the other hand, would be viewed as a “great” benefit. The codifiers are also divided on this matter. Rabbi Meir Abulafia held that under such circumstances one may injure oneself,^84^,^85^ while Maimonides held that one may not injure oneself,^86^ a ruling codified by Rabbi Joseph Karo.^87^

In view of this, the utilization of organs and body tissues for purely commercial purposes is not permissible. Similarly, it is prohibited to donate a kidney for research or industrial purposes if the benefit to the donor is purely financial. On the other hand, cutting the hair involves no injury, and it is therefore permissible to use hair for purely commercial reasons.

Blood donations fall somewhere between the examples discussed above. In drawing blood there is only minor discomfort. Is this similar to cutting hair, which is not considered an injury, and therefore permitted? Or is drawing blood more like kidney donations? Rabbi M. Feinstein tended to permit drawing blood for purely commercial reasons.^88^

Although one may not remove a kidney for mere financial benefit, one may surely remove it to transplant it for the prolongation of life. Even relief from suffering or improvement of the quality of life is considered to be of great enough benefit to justify the injury involved in removing a kidney.

When there is no prohibition of injury to the donor of an organ or tissue, does the donor have a right to demand payment? In principle, it would seem that the donor should have the same right to sell a kidney or blood as he has to sell his hair. But three points might restrict this right:

- As a rule, one should not accept payment to fulfill a commandment of the Torah.
- Society may legislate to prevent the exploitation of its poorer members.
- Informed consent and a firm decision to sell are necessary prerequisites for removal of an organ or tissue, and for transfer of ownership to the purchaser.

### PAYMENT FOR THE FULFILLMENT OF DIVINE COMMANDMENTS

In principle one may not insist on monetary compensation for teaching Torah.^89^,^90^ This is deduced from the well known Midrash (homiletic method of biblical exegesis; the term also refers to the whole compilation of homiletic teachings on the Bible), which compares the Almighty’s instruction of the Israelites in the days of Moses with the instruction of students by their teachers. Just as the Israelites were instructed without payment, so should students in every generation be instructed without charge. Nevertheless, according to Rabbi Jacob ben Asher it is permitted to accept payment for Torah learning.^91^

This principle is not limited to instruction in Torah. It encompasses the fulfillment of all commandments.^92^ Since healing is also a commandment of the Torah, in principle the healer may not demand payment for healing.^93^,^94^ it would apparently follow that one may not be reimbursed for donating an organ for life-saving purposes.

Although a healer may not demand compensation for his efforts in healing, he may request compensation for his expenses, his time, and any medications or devices which he gives the patient.^93^,^95^ in other words the fulfillment of a commandment does not require that the healer spend his own money for the patient.

It is obvious that the loss of an organ can, to some extent, be evaluated in terms of money. It can be concluded also from the Mishnah.^96^ The suffering involved in the removal of an organ is also measurable in financial terms – *“Tsa’ar”* in the terms of the Mishnah. Therefore, a donor has every right to demand compensation for a donated organ and for the suffering incurred by its removal, even when such an act is considered as a great mitzvah.

A reason presented for permitting midwives to receive compensation for the performance of their occupation on the Sabbath is: “because if they knew that they would not be paid, they might not come”.^97^ The same principle can be applied to *any* medical procedure of a life-saving nature.

Even if physicians were not allowed to receive compensation, there is a fundamental difference between the donor of an organ and a physician. A physician is charged with the commandment to heal. He cannot exempt himself from this obligation, and it may be argued that one who should not refuse rendering medical service has no claim to compensation.^98^,^99^ A donor, on the other hand, who is under no obligation to donate an organ, and may accordingly choose not to donate, has the right to claim compensation.

In summary, the general prohibition of compensation for fulfilling a commandment does not conflict with the right of a donor to demand and receive payment for his suffering and for the organs or tissues donated.

### EXPLOITATION OF THE POOR

It would seem that in a cruel world there is real danger of an organ market in which the affluent might purchase an organ from the poor. This is an example of exploitation of the poor by the rich. In order to prevent such legalized exploitation, it would be appropriate to introduce legislation regulating the sale of human organs and tissues (cf. Ta’amei Massoret ha-Mikra le-Rab Judah ha-Chasid, end Ki Teitsei; cf. Malbim on the Sifrei 134; cf. Ramban, Comm. on the Torah ibid.; Sefer ha-Chinnuch 580). Today, as we have no central halachic authority to legislate universally binding laws, rabbinic bodies have jurisdiction only in those locations which have accepted their authority.^100^–^102^

In summary, unless such a prohibition is legislated, we cannot prohibit the sale of organs for purely exploitative reasons, whether the donation of an organ may lengthen human life, or where it may improve the quality of life.

### INFORMED CONSENT AND COERCED SALE OF AN ORGAN

Secular Israeli law requires the patient’s signature on a consent form prior to surgery. The law stipulates the formula to be used. A physician is also required to sign a form certifying that he has explained to the patient everything contained in the form, that the patient fully understood, and that the patient signed the form in his presence.

The requirement that the patient fully understand the need for and the possible results of the surgery is impracticable in many cases. In fact, this requirement is fulfilled in only a minority of cases. Generally speaking, the patient has neither the medical knowledge nor the ability to weigh the matter seriously. The physician’s signature does not change these facts.

From the halachic point of view a surgical procedure which may save the patient’s life does not require his consent. But the removal of an organ to save another patient is different. In such a case consent of the donor is of great significance. Without explicit prior consent the donor might subsequently claim that consent was given in error and that he had never intended to allow removal of an organ or tissue from him.

It is doubtful if the profit-seeking donor always properly understands the medical issues involved in the donation. The donor’s need for money may lead him to ignore the medical consequences of his donation. As a result, the donor may be considered as not fully informed, and his consent might thus not be valid.

If a human organ is sold under coercion, the sale seems to be invalid since it fails to comply with one of the basic conditions of “meeting of the minds”. A donor who sells an organ because of urgent financial need is in a state of coercion. Payment for the coerced sale does not create a situation of consent unless the seller receives full value and loses nothing on the transaction.^103^,^104^

### THE OPINION OF THE GREAT CONTEMPORARY JEWISH SCHOLARS

“There is therefore no reason whatever to ban one who donates a part of his body from requesting and receiving payment for this. The amount of payment can be stipulated in advance and agreed between the donor and a member of the family of the recipient of the transplant ... Such payment, provided that it is within reasonable limits, need not be seen as unethical, since the donor undergoes physical and at times mental suffering, and as stated a person does not waive his rights to his organs ...

“At the same time, it must be pointed out that only *the donor himself* is allowed to receive payment for his donation. Any intermediary acting between the donor and the family of the recipient, whether an individual or an organization that undertakes to deal with the matter, must act strictly within the halacha, which regards this is as trouble that everyone is obliged to take, ‘restoring the [safe] body’ to its owner. They are therefore *forbidden* to accept any payment other than compensation for abandoning other work, as explained above regarding restoring lost property. This should certainly be embodied in statutory law, to save us from the danger of a trade in human organs developing.”^105^

Rabbi Shlomo Zalman Auerbach has a more consistent opinion. He wrote that in order to save life, both the donor as well as the mediator, are allowed to receive compensation.^106^ Nevertheless see Wigoda’s opinion on organ donation and organ sale.^107^

### PRACTICAL CONCLUSIONS OF SECTION 3:

- There is no halachic prohibition against receiving compensation for donated organs.
- Sale of an organ as a result of desperate financial distress may create a situation of coercion without full value being paid. Such a situation lacks “complete consent”, and the sale might be void.
- A donor’s incomplete understanding of the medical consequences of the removal of an organ may invalidate the sale.
- In light of the differences in various cases, the donation of organs for payment should be regulated and requires fully informed prior consent. This should eliminate exploitation on account of uninformed consent.
